# Resistance mechanisms to drug therapy in breast cancer and other solid tumors: An opinion

**DOI:** 10.12688/f1000research.10992.1

**Published:** 2017-03-17

**Authors:** Fedor Moiseenko, Nikita Volkov, Alexey Bogdanov, Michael Dubina, Vladimir Moiseyenko

**Affiliations:** 1 St Petersburg Academic University, St. Petersburg, 194021, Russian Federation; 2 St. Petersburg Clinical Research and Practical Center for Specialized Types of Medical Care (Oncologic), St. Petersburg, 197758, Russian Federation; 3 Peter the Great St. Petersburg Polytechnic University, St. Petersburg, 195251, Russian Federation; 4 The Petersburg Nuclear Physics Institute, Gatchina, 88300, Russian Federation

**Keywords:** cancer, breast cancer, chemotherapy, resistance

## Abstract

Cancer is an important contributor to mortality worldwide. Breast cancer is the most common solid tumor in women. Despite numerous drug combinations and regimens, all patients with advanced breast cancer, similarly to other solid tumors, inevitably develop resistance to treatment. Identified mechanisms of resistance could be classified into intra- and extracellular mechanisms. Intracellular mechanisms include drug metabolism and efflux, target modulations and damage restoration. Extracellular mechanisms might be attributed to the crosstalk between tumor cells and environmental factors. However, current knowledge concerning resistance mechanisms cannot completely explain the phenomenon of multi-drug resistance, which occurs in the vast majority of patients treated with chemotherapy. In this opinion article, we investigate the role of these factors in the development of drug-resistance.

Breast cancer is one of the most frequent cancers among solid tumors in women. Drug therapy is an important part of primary treatment for loco-regional breast cancer, and is a cornerstone of treatment in advanced disease ^1^. In contrast to the significant efficacy of first line chemotherapy, inevitably in subsequent lines a vast majority of patients develop drug resistance ^2^. Currently, knowledge concerning resistance to cytotoxic antineoplastic agents is based primarily and solely on separate mechanisms that underlie tolerance to single agents ^3– 10^. This approach, though it is experimentally verified, is not able to explain the resistance to multiple agents, which is not dependent on the mechanism of drug anticancer cation, and is either present at the beginning of treatment or formed during subsequent lines of therapy in all patients. Consequently, other universal complex mechanisms that allow tumor cells to escape inhibition by antineoplastic agents should exist and be revealed by now.

For localized stages, and particularly for breast cancer, elimination of tumor cells can be achieved by surgical excision or radical radiotherapy. Efficacy of these approaches does not depend on the heterogeneity of the tumor. Theoretically, administration of antineoplastic agents that interact with particular, sometimes not identified, mechanisms of tumor pathogenesis should also cause the death of all tumor cells, which would be equal to a cure. Due to various resistance mechanisms, described in detail below, drug therapy by itself rarely cures cancer, even in the case of such chemosensitive tumors as breast cancer. Malignant cells that survive primary treatment continue to evolve with appearance or overgrowth of a resistant clone population, which leads to progression and inevitably death of the patient. In these circumstances, identification of transforming mechanisms of resistance obtained by tumor cells might help to define optimal character, intensity and/or longevity of primary and consecutive treatment, which might achieve maximal eradication of tumor cells. This eradication by itself should decrease the clonal variability and influence the evolutionary potential of the tumor ^11^.

This paradigm is particularly important for hematologic malignancies. All clones are present in the bloodstream and/or bone marrow. Therefore, monitoring of the residual tumor burden has become possible with the introduction of new, highly sensitive molecular diagnostics, including direct sequencing, allele specific RT-PCR and digital PCR. For hematologic malignancies, it is essential to achieve a complete molecular response, which has been correlated with the longest time to disease progression. For example, in chronic myeloid leukemia complete cytogenetic and molecular response during the first three months of treatment is correlated with maximal survival and longest disease free interval ^12^.

Unfortunately, in contrast to hematologic malignancies in solid tumors, such as breast cancer, markers fitting the so-called “liquid biopsy” paradigm (i.e. circulating tumor cells and DNA) cannot always be found in biofluids, even in progressing advanced conditions. This peculiarity defines the necessity for the acquisition of histological, or at least cytological, samples from the primary tumor or metastatic site. As a good example, we can mention monitoring activating mutations in the biofluids of patients with non-small cell lung cancer. The identification of driver molecular alterations is currently possible, with a very high sensitivity ^13, 14^. However, even the most advanced technologies allow the correct identification of mutations in bioliquids in 6–7 out of 10 patients (for example, direct sequencing: 16.7–77.8%; PCR with enrichment: 4.7–49.3%; cobas ROCHE: 12.1%) ^15, 16^.

Furthermore, even achieving a complete clinical and radiologic response in tumors of a solid nature does not mean elimination of all tumor cells, as has been shown for preoperative treatment of rectal cancer or colorectal cancer metastases ^17, 18^. The same situation was also shown for perioperative treatment of breast cancer, where even one cell with epithelial markers found in bone marrow determines a significantly worse long-term outcome and risk of disease recurrence ^19^.

Despite a large number of identified mechanisms that may underlie resistance to conventional cytotoxic and targeted drugs, none of them can fully explain multidrug resistance, which is inevitably acquired by all patients with advanced breast cancer and other tumors. Among other examples of acquired resistance, one can mention the decrease in the longevity of second line treatment in comparison with first line ^8^. This decrease might be caused by the genetic heterogeneity that is a characteristic feature of all malignancies. This conception is illuminated in the GERCOR trial, where patients with inoperable colorectal cancer were randomized to two treatment groups. In group one, patients received FOLFOX as a first line and FOLFIRI as second and vice versa in group two. As a result no difference in overall survival was observed (21.5 versus 20.6 months; p = 0.99), but what is important is that no difference was observed in the progression free survival of first line (8.5 versus 8.0 months; p = 0.26) or second line (14.2 versus 10.9 months; p = 0.64) chemotherapy ^20^. Small differences were noticed in progression free survival of second line (4.2 versus 2.5 months, p = 0.003). Still the duration of the effect derived from first line was much larger than that of second line. This observation might be interpreted in the way that irrespectively of the initial regimen, the tumor mass at progression is composed of a clone with multidrug resistant features. This clone might have appeared during therapy or might have existed in a small proportion prior to the start of treatment. The latter can be illustrated with an example from NSCLC, when the T790M mutation that defines resistance to first generation TKI can be found in primary samples or may appear during TKI therapy ^14, 21^.

Furthermore, we can speculate that the appearance of a resistant clone or its presence at the initial tumor development might be probabilistic. To illustrate this idea, we can mention the N9741 trial, in which out of 1508 patients with inoperable colorectal cancer, complete radiologic response was seen in 62 patients. During consecutive follow up, 10/62 patients did not have disease progression and might be considered cured of metastatic disease ^22^. Thus, in combination with several circumstances, primary clones can be eradicated by primary chemotherapy and thus are not involved in the development of new resistant subclones.

Research aimed to define mechanisms of resistance are usually based on the determination of genotypic and/or phenotypic features that drive resistant clones, and a myriad of methods have been used, among them molecular, chemical and physical analysis. However, the most important avenue for resistance research might be the model by which resistance is created. There are two main directions to model the resistance to therapeutic agents: first, *in vitro* modeling of the interaction between tumor cells and active antineoplastic agent; and second, *in vivo* experimental systems, such as laboratory animals.

*In vitro* methods are historically the first type used. Interestingly, these methods were significant for antibiotics therapies, before it became necessary to use them for oncological purposes. Isolation and cultivation of a pathogenic microorganism beyond the host organism is used to define its sensitivity to antimicrobial agents, and describe phenotypes and molecular profiles, which are of essential importance for clinical decisions on treatment and for the development of newer agents.

This approach has been used for research in all malignant tumors. Immortalized cell lines and primary cell cultures have been successfully used to screen hundreds of components for antineoplastic activity and the definition of the mechanism of action of several therapeutic agents ^23, 24^. Unfortunately, despite numerous programs of investigation into resistance mechanisms in cell lines exposed to various doses and schedules of chemotherapeutic agents, a significant change in the understanding of these mechanisms has not occurred.

Firstly, unlike bacteria and other microorganisms, whose population in one host organism is limited with rarely more than one strain and evolution of resistance to antibiotics take place in several hosting organisms, evolution of malignant tumors is limited to the life of one host organism and is driven by the diversity of clones and genome instability. For this reason, isolation of a cell line or primary cell culture can hardly model the representative heterogeneous tumor cell population as it is inevitably accompanied by tumor cell dedifferentiation and loss of phenotypical heterogeneity. This observation might not *limit in vitro* drug testing programs, but significantly restricts resistance research potential.

Secondly, tumor cell cultures *in vitro* are usually deprived of microenvironment communication, which in some situations might be an essential mechanism for resistance generation and maintenance. Thirdly, tumor cell cultures are characterized by homogenous habitat conditions, for example there are no differences in the distance to supply blood vessels, which does not allow for model exposition to different drug concentrations at one time ^25^.

Nevertheless, programs conducted on cell cultures allow the determination of several mechanisms that might underlie resistance, or at least compromise the efficacy of various agents. Amidst them, one can mention various mechanisms, inlcuding mediating drug efflux (increased expression of ATP-binding cassette, including P-glycolprotein, multidrug-resistance-associated protein 1 and breast cancer resistance protein ^3, 26, 27^), increasing the expression of metabolic enzymes, deactivating cytotoxic drugs (CYP2C9*2), and modulating targets for cytotoxic drugs (increased expression pf beta-III-isoform of tubulin ^4^, increased expression of Tau ^6^, decreased expression of Top-II-alpha ^28, 29^). Unfortunately, patterns revealed once are rarely verified in consecutive series with the same conditions but different cell lines. Also, mechanisms identified as the primary mechanism in one series appear to be secondary or even nonsignificant in the others ^27^. As an example, we can mention an experiment where the efficacy of paclitaxel was compromised by different resistance mechanisms on one cell line exposed to different schedules of the drug ^29, 30^. Interestingly this appeared to be true also for the targeted agents, such as NSCLC with EGFR activating mutations that depended on the exposition dose of gefitinib developed either T790M or MET mediated resistance.

In conclusion, we suggest that the mechanism of multidrug resistance that inevitably develops during drug therapy of breast cancer, and other tumors of solid origin, have not yet been revealed. In our opinion the mechanism of resistance is most likely not directly related to drug metabolism or its target in the tumor cell.
