# Supplementary material for: Resistance mechanisms to drug therapy in breast cancer and other solid tumors: An opinion
Source: F1000Res. 2017 Mar 17;6:288. [Version 1] doi: 10.12688/f1000research.10992.1 (PMC5506585; doi:10.12688/f1000research.10992.1)
Supplement: Peer review history

# Peer review: Referee response for version 1

**DOI:** 10.5256/f1000research.11851.r24106

**Published:** 2017-07-10

**Authors:** Anna Herman-Antosiewicz

**Affiliations:** 1 Department of Medical Biology and Genetics, University of Gdańsk, Gdańsk, Poland

Recurrence and multidrug resistance is a major problem in cancer therapy. The authors discuss this important issue and it becomes clear that the first line treatment should be designed in such a way to eradicate all cancer cells, having in mind that at a time of diagnosis they are heterogenous and some of them are already drug resistant (in an otherwise naïve population).

 The authors highlight that numerous mechanisms responsible for resistance to therapy have been identified, mainly thanks to in vitro experiments. They also mention disadvantages of such an approach, including lack of original microenvironment which is unstable in its nature. That’s why, probably, none of the known mechanisms fully explain multidrug resistance. However, there is experimental evidence that microenvironment conditions during tumor development (pH or oxygen level changes) might drive genetic and phenotypic changes in cancer cells leading to their more aggressive character and multidrug resistance (for example, Taylor et al. (2015) ^1^ or Verduzco et al. (2015) ^2^. In my opinion, this aspect should be mentioned by the authors, as the tumor microenvironment might be a good target for an adjuvant treatment, also to prevent the recurrence of the more aggressive tumors.

 Minor comments:

- GERCOR trial results are incorrectly presented
- Misspelling: p1, line 11- should be: action (not cation)

I have read this submission. I believe that I have an appropriate level of expertise to confirm that it is of an acceptable scientific standard.

## References

[1] TaylorSSpugniniEPAssarafYGAzzaritoTRauchCFaisS: Microenvironment acidity as a major determinant of tumor chemoresistance: Proton pump inhibitors (PPIs) as a novel therapeutic approach. *Drug Resist Updat*.2015;23: 10.1016/j.drup.2015.08.004 69-78 10.1016/j.drup.2015.08.004 26341193

[2] VerduzcoDLloydMXuLIbrahim-HashimABalagurunathanYGatenbyRAGilliesRJ: Intermittent hypoxia selects for genotypes and phenotypes that increase survival, invasion, and therapy resistance. *PLoS One*.2015;10(3) : 10.1371/journal.pone.0120958 e0120958 10.1371/journal.pone.0120958 25811878PMC4374837

# Peer review: Referee response for version 1

**DOI:** 10.5256/f1000research.11851.r23679

**Published:** 2017-06-27

**Authors:** Phei Er Saw

**Affiliations:** 1 Post-Doctoral Fellow, Department of Anesthesiology, Harvard Medical School, Boston, MA, USA

The article is supposed to focus on the possible resistance mechanism to drug therapy in breast cancer. Yet, most literature review and examples cited are based on colorectal cancer, and none of the breast cancer trials are mentioned. The article is not articulated in a way to guide the readers point-to-point and rather scarce in consummating all the points systematically. 

 The author also did not clearly point out what are the current problems and resistance mechanism in breast cancer, what are the current approaches to overcome these problems and what is the possible outlook in overcoming drug resistance in breast cancer.

I have read this submission. I believe that I have an appropriate level of expertise to confirm that it is of an acceptable scientific standard, however I have significant reservations, as outlined above.

# Peer review: Referee response for version 1

**DOI:** 10.5256/f1000research.11851.r21105

**Published:** 2017-03-24

**Authors:** Alexey Tryakin

**Affiliations:** 1 Department of Clinical Pharmacology and Chemotherapy, N.N. Blokhin Russian Cancer Research Center, Moscow, Russian Federation

In this well-written mini-review the authors discuss important questions concerning mechanisms of tumor resistance which result to low concordance between *in vitro* and *in vivo* models.

**Minor comment**: Authors have made a mistake by citing GERGOR trial data (FOLFOX vs. FOLFIRI). They write: "As a result no difference in overall survival was observed (21.5 versus 20.6 months; p = 0.99), but what is important is that no difference was observed in the progression free survival of first line (8.5 versus 8.0 months; p = 0.26) **or second line (14.2 versus 10.9 months; p = 0.64)** chemotherapy ^20^. Small differences were noticed in progression free survival of second line (4.2 versus 2.5 months, p = 0.003). "

 PFS in second line was 4.2 vs. 2.5 months. However 14.2 vs 10.9 months was a second PFS (from the days 1 of 1-st line to the progression on 2-nd line). I suggest to omit data which I underlined.

I have read this submission. I believe that I have an appropriate level of expertise to confirm that it is of an acceptable scientific standard.
